# Remote biosensor for the determination of trypsin by using nanoporous anodic alumina as a three-dimensional nanostructured material

**DOI:** 10.1038/s41598-020-59287-7

**Published:** 2020-02-11

**Authors:** Mahmoud Amouzadeh Tabrizi, Josep Ferré-Borrull, Lluis F. Marsal

**Affiliations:** Departamento de Ingeniería Electrónica, Eléctrica y Automática, Universitat Rovira i Virgili, Avda. Països Catalans 26, 43007 Tarragona, Spain

**Keywords:** Diagnostic markers, Biosensors, Diagnostic markers, Biosensors

## Abstract

The determination of trypsin in the human real sample is a routine medical investigation to assess the pancreatic disease. Herein, we fabricated an interferometric reflectance spectroscopy based biosensor for the determination trypsin. For this purpose, urease and fluorescein 5(6)-isothiocyanate (FLITC) were immobilized on the nanoporous anodic alumina (NAA). The operation principle of the proposed biosensor is based on the change in the pH of the solution during the reaction of urease and urea and therefore change in the light-absorbing ability of FLITC in the presence of trypsin. The reaction of the urease enzyme with urea increased the pH of the solution because of producing ammonia. This increase in the pH of solution increased the light-absorbing ability of the immobilized FLITC on NAA and therefore the intensity of the reflected light from the NAA to the charge-coupled device detector decreased. In the presence of trypsin, the catalytic activity of immobilized urease on NAA decreased. This decrease in the activity of urease enzyme consequent on the decrease in the amount of the generated ammonia. Therefore, the immobilized FLITC on the NAA did not absorb more light and consciously, the intensity of the light reflected light into the detector increased. The proposed biosensor exhibited a good response to the concentration of trypsin in the range of 0.25–20 μg.mL^−1^ with the limit of detection of 0.06 μg.mL^−1^.

## Introduction

Millions of people in the world suffer from some form of pancreatic diseases^1^. According to world health statistics reports, pancreatic diseases id associated with a tremendous economic burden^2,3^. Due to reduce the economic losses caused by this disease and also to improve the public health, the fabrication of a device to diagnose this disease is important for every government. The routine laboratory test for pancreatic disease diagnosis is the determination of trypsin the human real sample. Trypsin is a serine protease with a potential role in pancreatic cancer that can cleavage the peptides on the carbonyl side of arginine and lysine amino acids^4^. In the acute pancreatitis patient that is a fatal disease, the concentration of trypsin reaches up to 84.4 µg.mL^−1^ in the urine sample that is so higher than its normal rage (115–350 ng.mL^−1^)^5^. Therefore, it is necessary to develop a simple, selective, sensitive, and low-cost sensor for trypsin detection. However, most of them are complicated, low sensitivity, time-consuming, and expensive method^6–10^. To overcome these problems, biosensors become more interested to detect trypsin^11,12^.

The biosensor is an analytical device which includes a transducer and a biomolecule (such as the enzymes, antibodies, and aptamers)^13–15^. Among the various biosensors that have been reported for the sensing of bio-targets, the optical biosensors are more interested because of their high sensitivity, small and lightweight, remote sensing ability, immunity to electromagnetic interference, capability for monitoring a wide range of chemical and physical parameters, and reliable operation^16–19^.

Up to now, several optical biosensors have been fabricated for the determination of trypsin^20–24^. Among them, the fluorescence biosensor based on quantum dots is more interested^25–30^. However, the fluorescence biosensor based on quantum dots are expensive methods and can not be suited for point-of-care testing. Therefore, the fabrication of a cheap but high sensitive optical biosensor for the determination of trypsin is needed.

To the best of our knowledge, the use of the interferometric reflectance spectroscopy (IRS) method for the fabrication of biosensor to detect trypsin has not been reported yet. The IRS is a contactless optical device that can be used for the determination of the various targets^19,31,32^. In this method, the beams of white light interfere with a reflective thin film then the partial beams of the light reflect the charge-coupled device (CCD) detector. Macroporous silicon (MPS) and nanoporous anodic alumina (NAA) are two common reflective thin films for the fabrication of the IRS based biosensor^19,33–35^. NAA has a lot of advantages such as high surface area, biocompatibility, easy functional ability, versatile, durable low-cost, and three-dimensional nanostructure platform^36–39^. According to the previous report, trypsin can cleave the amino acid chain from the carboxyl side (C-terminal side) of the amino acids lysine and arginine^40^. Therefore, the use of an enzyme that lysine and arginine amino acids play a major role in the function and structure of the enzyme and study the effect of trypsin on the catalytic activity of this enzyme, can be a novel method for the fabrication of biosensor for trypsin detection. Urease enzyme is a metalloenzyme that can catalyze the reaction of urea molecule into carbon dioxide and ammonia selectively. The amino acids lysine and arginine are two amino acids exist on the structure of urease enzyme. One of lysine amino acid bridges two Ni^2+^ ions (co-factor) to each other, playing a major role in the catalytic activity of the urease enzyme^41^. Up to now, several optical biosensors have been reported by using urease enzyme to detect the urea in the sample^42,43^. In these kinds of the optical-based biosensor, a pH-sensitive optical probe molecule such as fluorescein was used to study the generated ammonia from urea. In this paper, for the first time, an IRS method was used for the fabrication of an optical biosensor to detect trypsin as a model of protease proteins. To fabricate the IRS sensor, NAA, urease enzyme and fluorescein 5(6)-isothiocyanate (FLITC) were used as a reflective thin film, an enzyme that its activity can be changed by to protease proteins and an optical probe molecule, respectively. Compare with the fluorescence biosensor based on quantum dots, the proposed IRS based biosensor exhibited high analytical performance in terms of selectivity, sensitivity, stability, linear range and limit of detection in nearly every case.

## Experimental Section

### Reagents and chemicals

All chemicals were of analytical reagent grade and used without further purification. Double deionized (DI) water (18.6 MΩ) was used throughout. Aluminum (Al) discs of 15 mm diameter were obtained from Goodfellow. (3-aminopropyl) trimethoxysilane (3-APTES), oxalic acid, glutaraldehyde (GLA), phosphoric acid (H_3_PO_4_), sodium acetate (NaAC), chromium (VI) oxide (H_2_CrO_4_), perchloric acid (HClO_4_), cysteine, dopamine, glucose, nicotinamide adenine dinucleotide trypsin, urea, urease enzyme and were obtained from Sigma-Aldrich.

### Apparatus

Scanning electron microscopy (SEM) was performed with an FEI Quanta 600. Infrared spectra were obtained using a JASCO FT*/*IR*-*680 Plus Fourier transform infrared (FTIR) spectrometer. Raman scattering was performed on a Renishaw’s inVia Raman spectrometer using 514 nm laser source. The interferometric reflectance spectra were recorded using an AvaSpec*-*ULS3648 fiber optic spectrometer. EMITech K575X sputter coater was used to deposit 10 nm thick gold layer under vacuum at 30 mA for 1 min.

### The fabrication of NAA-NH-GLA-urease-FLITC

**Figure 1:**
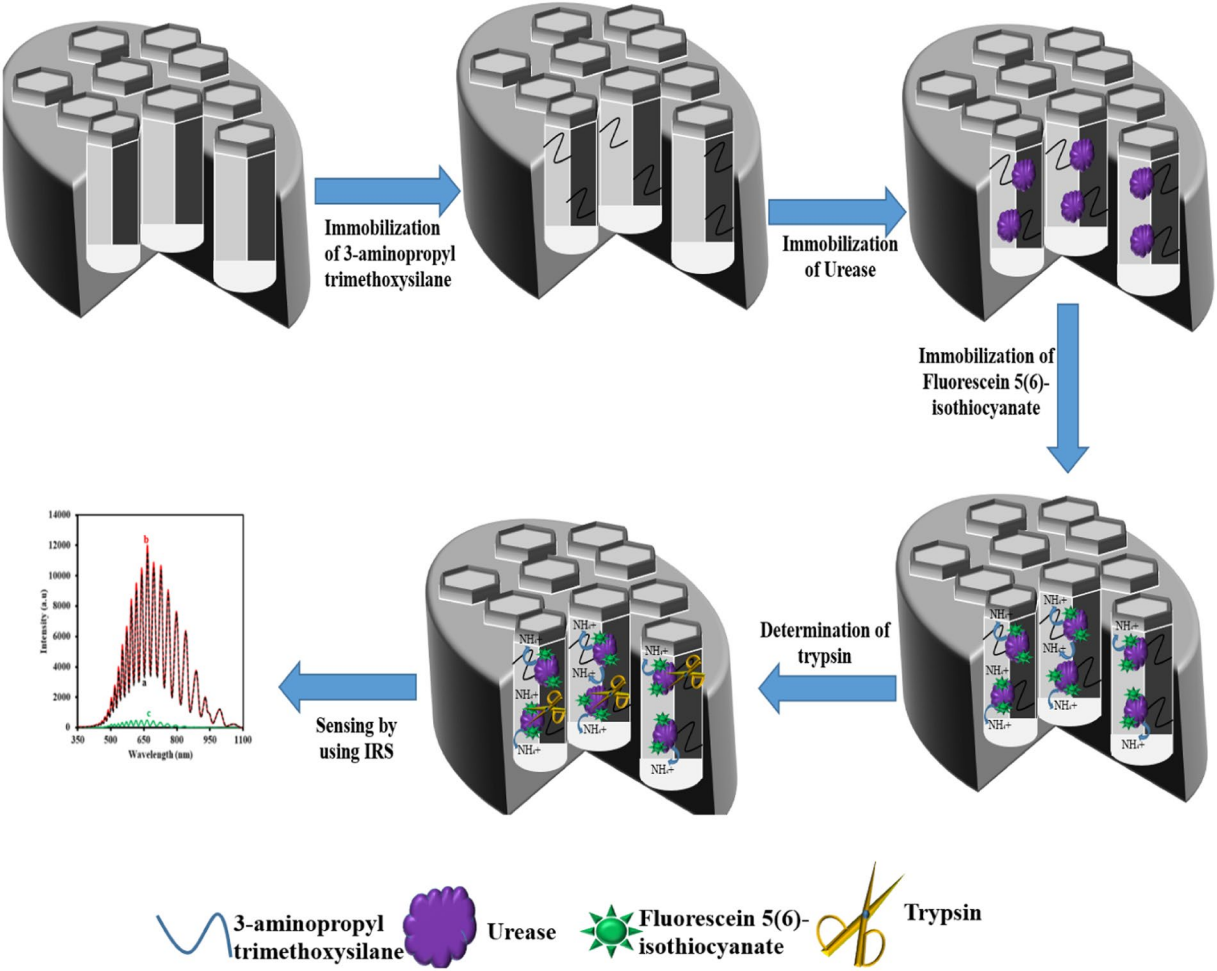
The schematic illustration for the fabrication of biosensor based on IRS.

NAA was prepared based on 2 steps electrochemical anodization process in oxalic acid solution under stirring condition^9,31,44,45^. The fabrication process is denoted in the electronic supporting material. The electrochemically fabricated NAA was then immersed into a 3-APTES solution (1% in ethanol/H_2_O 3:1) for 30 min under a nitrogen gas atmosphere to introduce amine groups inside of the pore of NAA. NAA-NH_2_ was then rinsed with deionized water for 1 min and dried under nitrogen gas flow. After that, the NAA-NH_2_ was dried under a nitrogen atmosphere at 100 °C for 2 h. After then, NAA-NH_2_ was immersed in a 2.5% GLA in phosphate buffer (PB) and stirred for 1 h. NAA-NH-GLA was then rinsed with water and dried under nitrogen gas flow to remove the unattached GLA. GLA is a crosslinker that can be used for the immobilization of enzymes to fabricate biosensors^46,47^. Subsequently, The NAA-NH-GLA was immersed in a urease solution (20 mg.mL^−1^, 0.1 M PB and pH 7.4) for 5 h under a stirring condition at 4 °C to immobilize urease enzyme on the NAA-NH-GLA. After rinsing with 0.1 M PB, the NAA-GLA-NH_2_-urease was immersed in a fluorescein iso-thiocyanate solution (1 mg.mL^−1^, 0.1 M PB and pH 8.4) for overnight at 4 °C to immobilize FLITC on the NAA-NH-GLA-urease. According to the previous report, the iso-thiocyanate (ITC) functional group can interact with sulfhydryl groups (–SH), amine (–NH_2_), and hydroxyl (–OH) functional groups^48^. Cysteine and lysine amino acids that have –SH and –NH_2_ functional groups, respectively and exist in the structure of the urease enzyme^49^. Therefore, the ITC function group of FLITC can interact with urease enzyme and immobilize on the NAA. After that, the NAA-GLA-NH_2_-urease-FLITC was rinsed with water several times to wash away any unattached FLITC. The NAA-urease-FLITC was stored at 4 °C in a refrigerator when not in use. The schematic representation for the fabrication of an IRS based biosensor is shown in Fig. 1.

### The sensing process of trypsin

The determination of trypsin was done in the three steps:

(1) 3 mM urea solution (0.1 M NaAC, pH 6.4) was transferred to cell reaction by using a peristaltic pump. After 30 seconds, the pump was turned off for 4 min and the signal of the biosensor was recorded after that. Before starting the second step, the biosensor was washed by pumping NaAC solution (0.1 M, pH 6.4) for 1 min. (2) The different concentration of trypsin solution (0.1 M NaAC, pH 9.4) was pumped to the measurement cell for 45 min. During this period of time, trypsin cleaved the urease enzyme form the arginine and L-lysine sites^39^. Then, NAA-NH-GLA-urease-FLITC was washed by NaAC solution (0.1 M, pH 6.4) for 5 min to remove the cleaved parts of the urease enzyme. (3) The urea solution was transferred with the same condition in the first step (0.1 M NaAC, pH 6.4, 3 mM urea, reaction time 4 min). The analyzed data showed that the recorded signal of the biosensor in the third step was lower than in the first step. The reasonable explanation is that the cleaved urease by trypsin could not catalyze urea as much as the first step and then the amount of the generated ammonia was lower than before. Consequently, the immobilized FLITC could not absorb the white light in this low pH solution as much as in the first step. Therefore, the intensity of the white light was detected by the CCD detector was higher than in the first step.

## Results and Discussion

### The surface morphology of the prepared NAA

**Figure 2:**
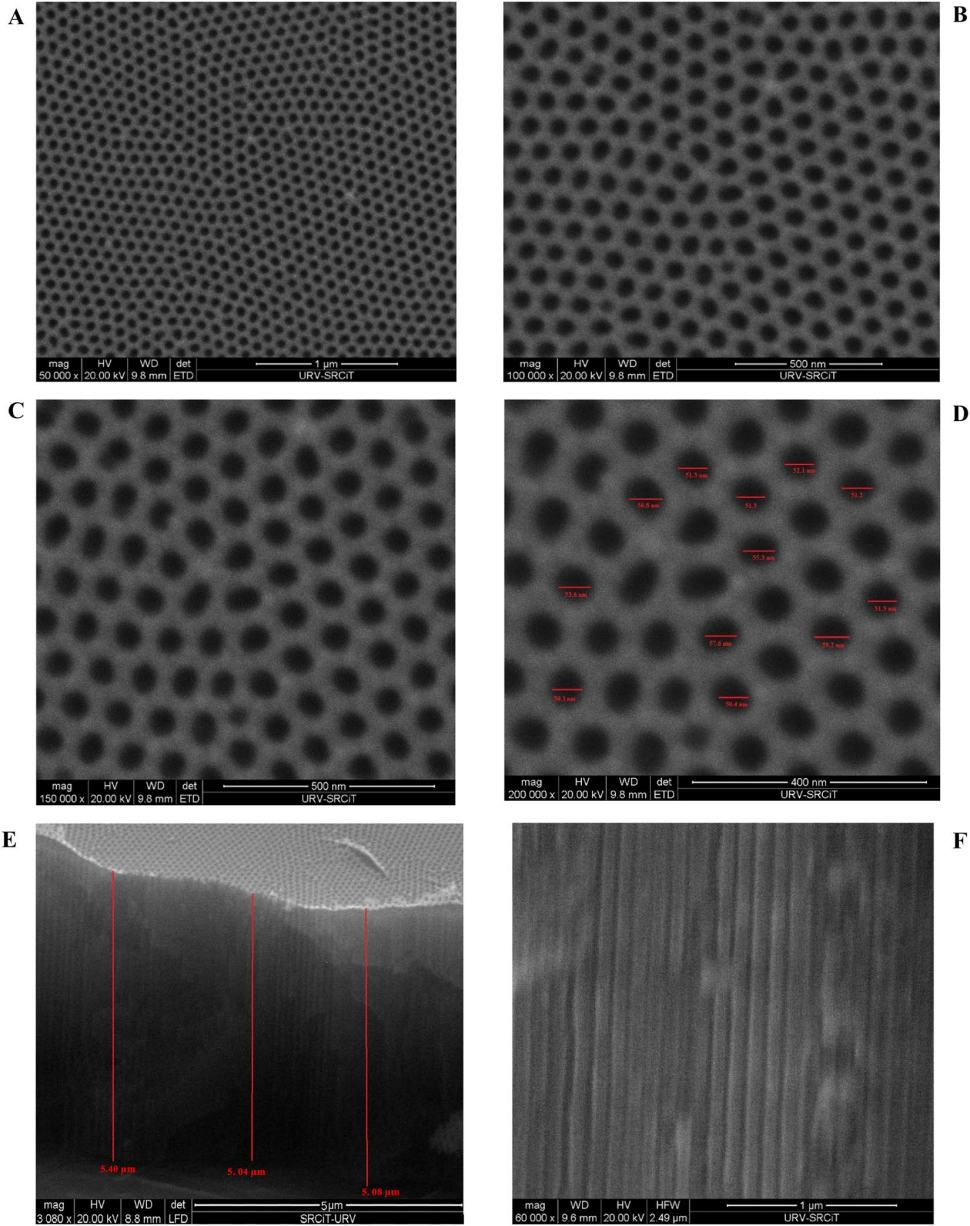
SEM images of NAA: (**A**–**D**) top views and (**E**,**F**) cross*-*section view.

The morphological characterization of the NAA was examined by SEM (Fig. 2A–F). As shown in these figures, the surface of the NAA has a hexagonal multi-pore structure. The average pore diameter and pore length of NAA are 54 nm (with a standard deviation of 2.1 nm) and 5.17 µm (with a standard deviation of 0.07 µm), respectively. According to the previous report^50^, the best Fabry-Perot fringe spectrum of the NAA would be obtained as the pore length/pore diameter aspect ratio was around 90 to100. Since the aspect ratio of the fabricated NAA for the determination of trypsin was 95.7, therefore the biosensor signal would be high.

Also, the pore diameter of the fabricated NAA is bigger than trypsin enzyme (3.8 × 3.8 × 3.8 nm)^51^, therefore, trypsin enzyme can get in the nanopore of NAA easily.

### Characterization of NAA-NH-GLA-urease-FLITC

FTIR and Raman spectroscopy are commonly characterization techniques of materials.

**Figure 3:**
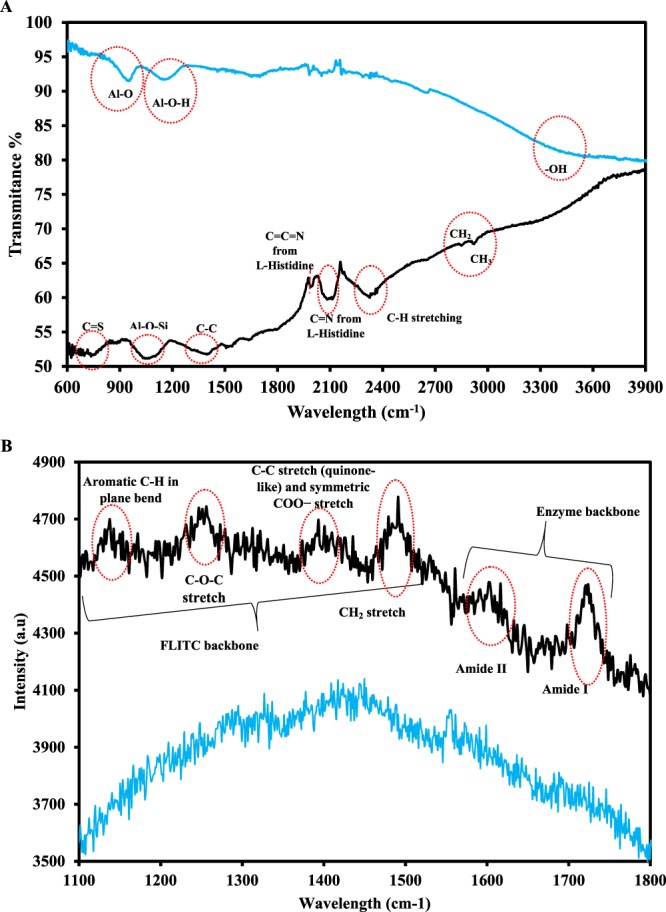
(**A**) FTIR and (**B**) Raman NAA-urease-FLITC spectrums.

FTIR spectrum of NAA (Fig. 3A (curve a)) exhibited a band at 3408 cm^−1^ due to the O–H stretching mode, and two absorption bands at 1159 cm^−1^ and 955 cm^−1^ due to the Al-O-H and the Al-O modes of boehmite, respectively^52^. After the immobilization of urease-FLITC on NAA, the FTIR spectrum changed noticeably (Fig. 3A (curve b)).

As shown in Fig. 3A (curve b), an absorption band at 736 cm^−1^ due to the –C=S stretching of the FLITC is clearly seen. The other characteristic absorption bands a band at 1058 cm^−1^ due to the −Al–O–Si, a band at 1394 cm^−1^ due to the –C–C stretching, a band at 1991 cm^−1^ due the –C=C=N (from L-histidine of urease enzyme), a strong band at 2112 cm^−1^ due to –C=N (from L-histidine of urease enzyme), a strong band at 2324 cm^−1^ due to the –C–H stretching, a band 2850 cm^−1^ due to the −CH_3_, and a band at 2920 cm^−1^ due to the –CH_2_ are also clearly seen in this figure^53,54^.

Figure 3B shows a typical Raman spectrum of NAA (a) NAA-NH-GLA-urease-FLITC (b). As can be seen, NAA has not any band in this wavelength. However, several bands were observed in Raman spectrum of NAA-NH-GLA-urease-FLITC. The band in the range of 1140 cm^−1^ are attributed to the aromatic –C–H in-plane bend, a band at 1250 cm^−1^ due to the –C–O–C stretching mode and a band at 1414 cm^−1^ due to the –C–C stretching of quinone and symmetric carboxylic (–COO^−^) stretch mode from FLITC and a band at 1600 cm^−1^ due to the amide II stretching, and a band at 1723 cm^−1^ due to amide I of urease enzyme^53,54^. These results demonstrate that FLITC and urease enzyme was immobilized on the NAA, successfully.

### The optical characterization of the biosensor

**Figure 4:**
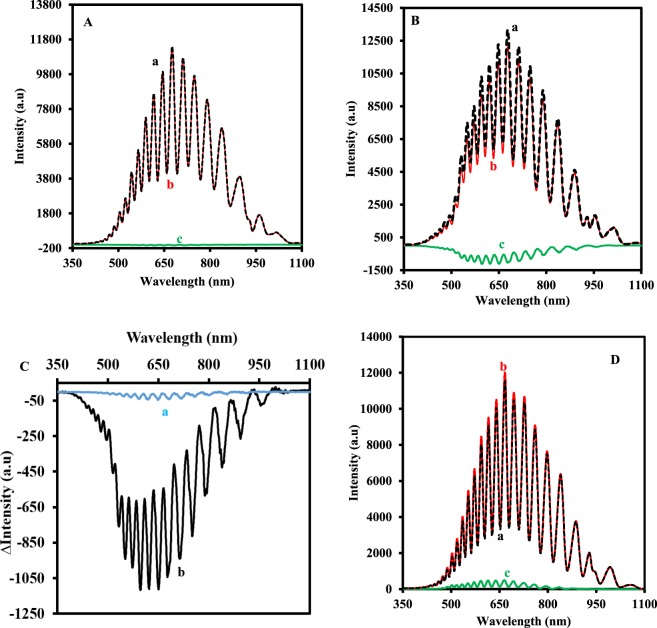
(**A**) IRS of the NAA-urease and (**B**) NAA-urease-FLITC in the absence (a) and presence (b) of 3.0 mM urea and (c) the difference between two interference spectrums. (**C**) ∆IRS of the NAA-urease (a) and NAA-urease-FLITC (b) in a solution (0.1 M NaAC, pH = 6.4) containing 3 mM urea. (**D**) IRS of the NAA-urease-FLITC in the absence (a) and presence (b) of 20.0 μg.mL^−1^ trypsin and (c) the difference between two interference spectrums.

Figure 4A shows the typical the interference spectra of NAA-NH-GLA-urease (A), NAA-NH-GLA-urease-FLITC (B) in the absence (a) and presence (b) of 3 mM urea (0.1 M NaAC, pH 6.4). The spectrums (c) in these figures relate to the difference of spectrum (a) and (b) that obtained from the subtraction of spectrum (a) from the spectrum (b). As can be seen, the sensitivity of NAA-NH-GLA-urease to 3 mM urea is low. However, after the immobilization of FLITC as a pH photo-probe molecule, the response of NAA-NH-GLA-urease-FLITC is dramatically higher than NAA-NH-GLA-urease. This difference in the signal of the NAA-NH-GLA-urease and NAA-NH-GLA-urease-FLITC to 3 mM urea was shown in Fig. 4C. The reasonable explanation is that the reaction of the urease enzyme with urea increased the pH of the solution because of producing ammonia. This increase in the pH of solution increased the light-absorbing ability of the immobilized FLITC on NAA and therefore the intensity of the reflected light from the NAA to the charge-coupled device (CCD) detector decreased.

Also, Fig. 4D shows the IRS of the NAA-urease-FLITC in the absence (a) and presence (b) of 20.0 μg.mL^−1^ trypsin and (c) the difference between two interference spectrums. As can be seen, the intensity of the IRS increased in the presence of trypsin due to urease enzyme digestion and deactivation, the pH of the solution during the reaction of urease and urea did not increase and then FLITC did not absorb the interfered with light. Hence, the intensity of the reflected light to the CCD detector increased.

### Optimization of effective parameters on the response of the biosensor

The pH of the solution and incubation time were optimized. The respective figures are given in the electronic supporting material (Fig. S1). The following experimental conditions were found to give the best results: (a) pH of 6.4 the solution, (b) interaction time of 4 min for urea (3 mM) and (c) pH of 9.4 the solution (d) 45 min for trypsin (3 μg.mL^−1^). As can be seen, the optimum pH of the solution for the determination of urea is 6.4. The reasonable reason to explain this phenomenon is, the activity of urease enzyme decreased in the pH less than 6.4 and the change in the absorbance intensity of FLITC from pH 6.4 to alkaline pH is high. Therefore, the immobilized FLITC absorbed more white light and the response of NAA-NH-GLA-urease-FLITC to urea in pH 6.4 solutions is higher than the other pH solution. Also, the average pH of the urine sample is about 6. Therefore, pH 6.4 was chosen as the optimum pH of the solution. Since the proposed biosensor is a pH-based biosensor, the determination of the analyte should be done at the solution that its pH buffering capacity is lower than 6.4. Since the pH buffering capacity of phosphate solution is in the range of pH 6.2–8.2, NaAC was used as the electrolyte of solution that its pH buffering capacity is in the range of 3.8–5.8. Figure S1B shows the effect of time on the response of biosensor to urea. As can be seen, the response of biosensor to urea increased rapidly with increasing time up to 4 min and remained unchanged at longer reaction times, suggesting that the reaction has reached the saturation level. Also, Figure S1C shows the effect of pH solution to the response of the proposed biosensor in the presence of 2 μg.mL^−1^ trypsin. As shown, the trypsin has a high activity to cleave the urease enzyme in the pH 9.4. The result has a good agreement with the previous report^55^. The reasonable reason to explain this phenomenon is the structure of the enzyme is changed and the active site is distorted in the higher than 9.5 and pH solution and the activity of trypsin decreased. Figure S1D shows the effect of time on the response of biosensor to trypsin. As can be seen, the response of biosensor to trypsin increased rapidly with increasing time up to 45 min and then remained unchanged at longer reaction times, suggesting that the reaction has reached the saturation level.

### Detection of trypsin

**Figure 5:**
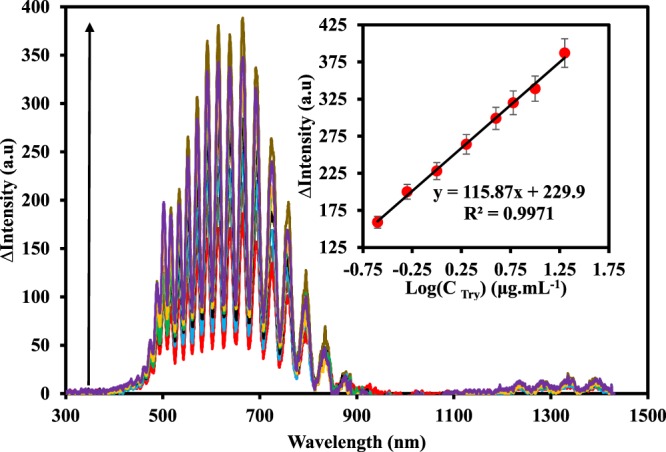
IRS of the NAA-urease-FLITC in a solution (0.1 M NaAC, pH = 6.4, 3 mM urea) containing different amounts of trypsin (0.25, 0.5, 1.0, 2.0, 4.0, 6.0, 10.0, and 20.0 μg.mL^−1^ from outer to inner). Inset: the corresponding calibration curve of the proposed IRS based sensor. Error bars represent standard deviations of four repeated experiments.

Under the optimized experimental conditions, the proposed biosensor was employed for the determination of trypsin (Fig. 5). It can be seen that the signal increase with increasing urea. As indicated in the inset of Fig. 5, a linear relationship between the logarithm of trypsin concentration (C_tryp_) and the intensity of the IRS was observed in the linear dynamic range (LDR) from 0.25–20.0 µg.mL^−1^ with a regression equation of ∆Intensity = 115.87 log C_tryp_ (µg.mL^−1^) + 229.9 and a correlation coefficient of 0.9971. The limit of detection (LOD) was 0.06 µg.mL^−1^ (at 3σ/S). The LOD of the proposed method is superior to the previously reported IRS based sensor for trypsin sensing (25 µg.mL^−1^)^56^.

Table 1 compares the LDR and the LOD of the proposed IRS based biosensor for trypsin determination with some of the optical biosensing methods such as nanoclusters^26,27^ and quantum dots^25,28^. As can be seen, although the LOD of CuNCs-protamine is lower than the proposed IRS based biosensor, however, it´s LDR is not wider than it. In addition, the fabrication and then purification of the functionalized nanoclusters and quantum dots are a very complicated, costly and time-consuming process.

**Table 1:** Comparison of the analytical performance of the proposed IRS based biosensor with some of the optical biosensors.

Biosensor	Method	LDR	LOD	Ref
GQD-BSA-FLTC	FL	0.7–6 μg.mL^−1^	0.7 μg.mL^−1^	^25^
BSA–Au NCs	UV–vis	0.9–1000 μg.mL^−1^	0.6 μg.mL-1	^26^
CuNCs-protamine	FL	0.1–1000 ng.mL^−1^	0.048 ng mL^−1^.	^27^
Mn:ZnSe doped quantum dots*-*Arg_6_	FL	0.1–12.0 μg.mL^−1^	0.04 μg mL^−1^	^28^
F-BSF/PFPS	FL	0.2–2.0 μg.mL^−1^	0.2 μg.mL^−1^	^29^
Fullerene-FLTC	FL	0.44–70.4 μg.mL^−1^	0.04 μg.mL^−1^	^30^
Palladium nanoparticles	FL	0.2–8 μg.mL^−1^	0.18 μg.mL^−1^	^57^
Cyt C in the presence of H_2_O_2_–and thiamine	FL	0.5–20.0 μg.mL^−1^	0.125 μg.mL^−1^	^8^
P-Al in the presence of morin	PL	40–300 μg.mL^−1^	40 μg.mL^−1^	^58^
P-Al	PL	—	100 μg.mL^−1^	^9^
NAA-Gelatin	IRS	0.1–1 mg.mL^−1^	25 μg.mL^−1^	^53^
NAA-urease-FLITC	IRS	0.25–20.0 μg.mL^−1^	0.06 μg.mL^−1^	This work

GQD-:BSA: Graphene quantum dots-Bovine serum albumin; CuNCs: Copper nanoclusters; BSA-AuNCs: Bovine serum albumin-nanoclusters; F-BSF: Benzenesulfonyl fluoride; FL; Fluorescence spectroscopy; Arg_6_: six arginine residues; Cyt C: Cytochrome c; P-Al: Anodic porous alumina PL: Photoluminescence

Furthermore, the proposed IRS based biosensor is not only a single-step measuring method but also is a remote sensing, lightweight, and low-cost device. Therefore, the analytical performance of the proposed optical biosensor in most cases is better than the optical biosensors, by considering all these aspects.

### The reproducibility stability and selectivity of the NAA-NH-GLA-urease-FLITC

The NAA-NH-GLA-urease-FLITC exhibited good reproducibility in the detection of trypsin. The relative standard deviation (RSD) for trypsin was founded 6.9% for over 5 repeated measurements of 3.0 µg.mL^−1^ trypsin. When not in use, the biosensor was stored at 4 °C in a refrigerator for 3 weeks, its signal of biosensor decreased by approximately 8.1%. The effect interfering compounds were also studied on the response of biosensor (Fig. S2). No interference was observed with 0.1 mg.mL^−1^ of cysteine, dopamine, glucose, nicotinamide adenine dinucleotide, and common cations and anions (0.15 mg.mL^−1^ of Na^+^, Al^+3^, K^+^, Cl^-,^ NO_3_). It clearly proves that the biosensor has high selectivity trypsin.

The Inhibition efficiency of the biosensor by trypsin also studied (Fig. S3). The IC_50_ value was obtained from the fitting curve was 12.4 µg.mL^−1^.

### Analytical application of the biosensor

The proposed biosensor was also employed for the determination of trypsin in the urine. Since the working range of the proposed biosensor was up to 20.0 µg.mL^−1^ and the concentration of trypsin in the patient’s urine is high (84.4 µg.mL^−1^), therefore, the urine sample should be diluted to get trypsin concentration into calibration range. For this purpose, 18.2 µL of a human urine sample (urea concentration was 192.0 mM) was diluted with 981.8 µL NaAC (0.1 M, pH 6.4) to decrease the concentration of the urea to 3.5 mM. Then, 858.1 µL of the diluted human urine sample was added to 141.9 µL of trypsin (20.0 µg.mL^−1^, 0.1 M NaAC, pH 6.4). The concentration of urea and trypsin reached 3.0 mM and 2.0 µg.mL^−1^, respectively. Finally, the sample was pumped to the analytical cell to determine. The obtained recoveries for 2.0 µg.mL^−1^ trypsin was 86%.

## Conclusion

This study introduced a biosensor based on the IRS for the determination of trypsin. To fabricate this biosensor, the urease enzyme and FLITC were immobilized on the pores of NAA as a urease enzyme and FLITC were used as an enzyme that its activity can be changed by to protease proteins and an optical probe molecule, respectively. The immobilized urease enzyme catalyzed the hydrolysis of urea to ammonia, letting to the pH-induced change in the light-absorbing ability of FLITC. The catalytic ability of the immobilized urease enzyme in the presence of trypsin as a bio-inhibitor changed. This change has a logarithmic relationship with the concentration of trypsin in the range 0.25–20.0 µg.mL^−1^. The proposed biosensor was also applied in the determination of trypsin in a human urine sample. The analytical performance of the proposed IRS biosensor was better than the fluorescence biosensor based on quantum dots.

## Supplementary information

Supporting data.
