# Supplementary material for: Remote biosensor for the determination of trypsin by using nanoporous anodic alumina as a three-dimensional nanostructured material
Source: Sci Rep. 2020 Feb 11;10:2356. doi: 10.1038/s41598-020-59287-7 (PMC7012875; doi:10.1038/s41598-020-59287-7)
Supplement: Supplementary file 1 — Supporting data. [file 41598_2020_59287_MOESM1_ESM.docx]

**Remote biosensor for the determination of trypsin by using nanoporous anodic alumina as a three-dimensional nanostructured material**

Mahmoud Amouzadeh Tabrizi, Josep Ferré-Borrull, [Lluis F. Marsal](https://www.sciencedirect.com/science/article/pii/S0925400517320701#!)*

Departamento de Ingeniería Electrónica, Eléctrica y Automática, Universitat Rovira i Virgili, Avda. Països Catalans 26, 43007 Tarragona, Spain

⁎ Corresponding author

E-mail address: lluis.marsal@urv.cat

The fabrication of NAA

Briefly, Aluminium (Al) disc of 15 mm diameter was first cleaned in ethanol and water (50: 50) under ultra-sonication for 5 min and then in acetone for 5 min to remove any pollution on the surface of Al. To electro-polish of Al disc, the freshly cleaned Al disc was attached to the electrochemical cell. After that, Al disc was anodized at 20 V in an ethanol solution containing HClO_4_ (25%) for 10 min. Subsequently, Al disc was washed several times and dried under compressed airflow to remove all residual HClO_4_. The first step anodization was then carried out in 0.3 M Ox at 40 V and 5 °C for 20 h. The Al disc was then rinsed by water and ethanol for 5 min and dried under compressed airflow. Afterward, Al disc was dissolved by wet chemical etching in a solution containing 0.4 M H_3_PO_4_ and 0.2 M H_2_CrO_4_ for 3 h at 70 °C. The Al disc was then rinsed by water and ethanol for 10 min and dried under compressed airflow. Afterward, the second step anodization was performed under the same conditions as first anodization and it was done until a total charge of 20 C was passed to obtain a layer of 5 μm thickness. The pore widening was done by wet chemical etching by immersing in 5% H_3_PO_4_ for 20 min. To provide high fidelity interferometric reflectance of NAA, the surface of NAA was first coated with 10 nm thick gold layer under vacuum at 30 mA for 1 min using an EMITech K575X sputter coater. After that, NAA was immersed into a 3.0 M H_2_O_2_ solution (T = 70 °C) for 1 h to removes any organic contaminants from the surface and actives the native hydroxyl groups of NAA. Then, NAA was washed with water for 30 s and dried under nitrogen gas flow.

| **A** | **B** |
| --- | --- |

| **C** | **D** |
| --- | --- |

**Figure S1.** (A) Effect of pH of the solution and time (B) on the response of biosensor to 3 mM urea. (C) Effect of pH of the solution and time (D) on the response of biosensor to 3 µg.mL^-1^ trypsin. Error bars represent standard deviations of four repeated experiments.

**Figure S2.** The selectivity of the proposed biosensor to 3.0 µg.mL^-1^ trypsin in the presence of 50 times the excess (0.15 mg.mL^-1^) of cysteine, dopamine, glucose, nicotinamide adenine dinucleotide, Na^+^, Al^+3^, K^+^, Cl^-,^ NO_3_.

**Figure S3.** The inhibition efficiency of the biosensor. The IC_50_ values were obtained from the fitting curve.
